# Crystal structures of RidA, an important enzyme for the prevention of toxic side products

**DOI:** 10.1038/srep30494

**Published:** 2016-07-26

**Authors:** Xiwen Liu, Jianhua Zeng, Xiaolei Chen, Wei Xie

**Affiliations:** 1 State Key Laboratory for Biocontrol, School of Life Sciences, The Sun Yat-Sen University, 135 W. Xingang Rd., Guangzhou, Guangdong 510275, People’s Republic of China; 2 Center for Cellular & Structural biology, The Sun Yat-Sen University, 132 E. Circle Rd., University City, Guangzhou, Guangdong 510006, People’s Republic of China

## Abstract

The YjgF/YER057c/UK114 family proteins are highly conserved across all three domains of life, and most of them currently have no clearly defined biological roles. *In vitro*, these proteins were found to hydrolyze the enamine/imine intermediates generated from serine or threonine, and were renamed Reactive Intermediate Deaminase A (RidA). RidA was recently discovered in *Arabidopsis thaliana,* and by deaminating the toxic enamine/imine intermediates, it prevents the inactivation of many functionally important pyridoxal 5′-phosphate (PLP)-containing enzymes in plants such as branched-chain aminotransferase BCAT (IlvE). In this study, we determined the crystal structure of *Arabidopsis thaliana* RidA in the apo form, as well as RidA complexed with the ligand pyruvate. RidA forms the trimeric, barrel-like quaternary structure and inter-subunit cavities, and resembles most RidA family members. Each pyruvate molecule binds to the interface between two subunits, and the recognition of pyruvate is achieved by the interactions with R165 and T167. From sequence alignment and structural superposition, we identified a series of key residues responsible for the trimer assembly, whose importance was confirmed by enzymatic assays. This study provides structural insight into RidA functions in plants.

The YjgF/YER057c/UK114 proteins were named after proteins YjgF, YER057c, and UK114, which were found in *Escherichia coli*, yeast, and goat respectively. These small proteins (~15 kDa) are found across all three domains of life with a signature of 6–9 conserved amino acids, and to date, hundreds of putative members have been identified by bioinformatic analyses. They appear to play important roles in highly diverse biological processes, many of which are poorly understood despite their universal presence^1^,^2^,^3^. Studies have suggested that they remove the reactive metabolites that could otherwise inhibit key metabolic enzymes. In bacteria and yeasts, YjgF/YER057c/UK114 proteins are associated with distinct pathways such as the isoleucine, thiamine, and purine biosynthesis^4^,^5^,^6^,^7^. YjgF protein from *Salmonella enterica* was found to deaminate the enamine/imine intermediates generated by pyridoxal 5′-phosphate (PLP)-dependent serine/threonine dehydratases, and was renamed RidA (reactive intermediate/imine deaminase A)^8^. Not only can these enamine/imines inactivate PLP enzymes by acting on the PLP cofactor^9^,^10^, but also they form Michael adducts with cysteines (reviewed by reference 11). Therefore the enamine/imine intermediates cause significant cellular damage if allowed to accumulate, and RidA is responsible for the clearance of these reactive species^12^. Other RidA functions including Hsp90-like chaperone activity^13^, ribonuclease activity^14^, photosynthesis as well as chromoplastogenesis^15^ have also been reported.

In 2014, RidA proteins were identified in *Arabidopsis thaliana* and maize^16^. *Arabidopsis thaliana* RidA (AtRidA) is targeted to chloroplasts. By converting the reactive enamine/imines to harmless 2-oxoacids, RidA preempts damage to BCAT3 and ensures that the isoleucine biosynthesis can proceed.

A recent phylogenetic analysis suggested that RidA could be divided into seven subfamilies (Rid1 to Rid7) besides the archetypal RidA subfamily. These subfamilies are generally found in bacteria, and coexist with each other in the same organism including RidA. The Rid1 to Rid3 subfamilies contain a conserved arginine residue known to be essential for the imine-hydrolyzing activity in the archetypal RidA and Rid2 subfamilies. By contrast, the Rid4 to Rid7 subfamilies appear not to hydrolyze imines, and their functions are yet to be identified^17^.

A few crystal structures have been reported for the YjgF family proteins (for example, PDB codes 1QD9, 1X25, 1QU9, 2UYN and 1ONI)^18^,^19^,^20^,^21^,^22^. These proteins share similar homotrimeric biological structures, and they display a barrel shape with a deep cavity in the trimer. Additionally, three clefts are formed at the interfaces of adjacent monomers, which are considered to be the putative ligand-binding sites^18^. In 2007, the crystal structures of *Escherichia coli* TdcF in complexes with a variety of ligands including 2-oxobutanoate were solved, which provided great insight into the substrate recognition mechanism of RidA family of proteins^22^. Low molecular-weight metabolites with a carboxylate group such as 2-oxobutanoate, propionate, serine, acetate, and benzoate are capable of binding at the clefts between the two monomers^18^,^20^,^22^. The interactions with 2-oxobutanoate involve a bi-dentate salt bridge with R105, and the keto group makes two additional hydrogen bonds with C107 and E120 respectively.

In this study, we determined the crystal structures of apo-AtRidA and its complex with pyruvate, and analyzed its structural basis for ligand recognition. AtRidA retains the typical domain as observed in many other RidA members. Using sequence alignment and structural superposition methods, we identified critical residues for trimer assembly as well as catalysis, and their importance to enzyme functions was evaluated by size-exclusion chromatography analyses and enzymatic assays.

## Results and Discussion

### Overall structure of apoenzyme and comparison with homologous proteins

**Figure 1:**
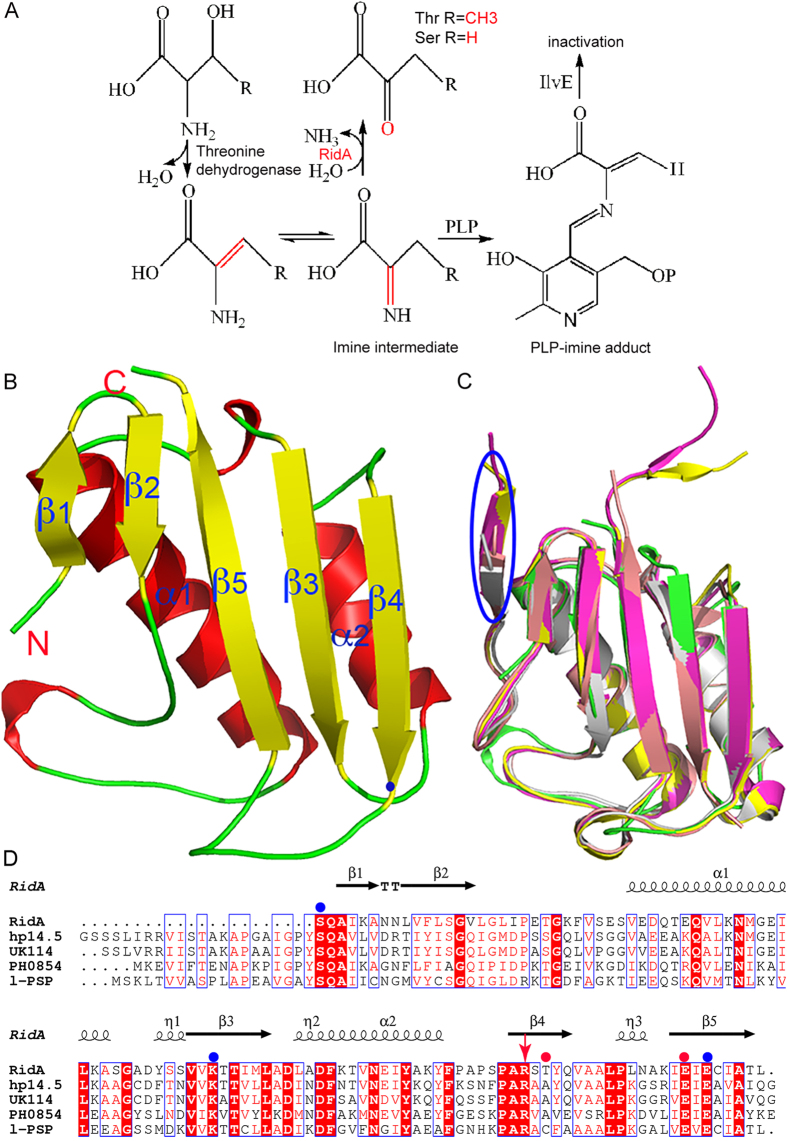
The enzymatic pathway that RidA participates and its overall structure. (**A**) The metabolic pathway involving AtRidA. (**B**) The structure of the RidA monomer. The major secondary structural elements, the N-, and C-termini of RidA are labeled. The helices, strands and loops are colored in red, yellow and green respectively. (**C**) The structure overlay of RidA and structural homologs. AtRidA (PDB code 5HP7, green), *Homo sapiens* hp14.5 (PDB code 1ONI, magenta), *Capra hircus* UK114 from (PDB code 1NQ3, yellow), *Pyrococcus horikoshii* PH0854 (PDB code 2DYY, dirty violet), and *Entamoeba histolytica* l-PSP (PDB code 3M4S, gray) respectively. The extra strand present in other homologs is circled by the blue oval. (**D**) Multiple sequence alignment of RidA homologs as in (**C**). The secondary structure of RidA is drawn on the top. Identical residues in sequences are on a red background, and similar residues are in red. Critical residues involved in the trimer formation are indicated by the solid blue circles, while residues for ligand binding are represented by the solid red circles. R165 is indicated by the red arrow.

The N-terminus of RidA is predicted to harbor a disordered region, which may function as a transit peptide targeting RidA to chloroplasts^16^. To obtain crystals of the apoenzyme, we created several constructs with different starting sites within the sequence. The current crystal structure was produced by the construct starting from S68 (named AtRidA-S68), and it diffracted to a resolution of 2.0 Å. In the final model, residues S80-L187, as well as 85 water molecules are resolved. The model displays good geometry, with 98.1% and 1.9% residues falling into the Ramachandran-favored and allowed regions respectively. Each asymmetric unit contains a single monomer, with a Matthew’s coefficient of 1.85 and an estimated solvent content of 33.7%. The model shows a mixed β-sheet packed against two long α-helices (Fig. 1B). In the β-sheet, the strands are antiparallel except for the outermost strand β4, which is parallel to β3. An extended loop L91-E110 is located between β2 and α1.

A DALI search from the PDB database retrieves several close structural homologs for RidA, and they are the acid-soluble protein hp14.5 from *Homo sapiens* (PDB code 1ONI)^20^, the tumor associated antigen UK114 from *Capra hircus* (PDB code 1NQ3)^23^, the putative translation initiation inhibitor PH0854 from *Pyrococcus horikoshii* (PDB code 2DYY, unpublished), and a putative endoribonuclease l-PSP from *Entamoeba histolytica* (PDB code3M4S, unpublished) respectively. AtRidA-S68 shares ~54% sequence identity with its closest structural neighbor hp14.5, with a root-mean-square deviation (RMSD) of 0.71 Å over 107 Cα atoms (Fig. 1C,D). Additionally, AtRidA is highly similar to other RidA family members in structure, and their overall structures are conserved. The most obvious difference is that AtRidA lacks an extra antiparallel strand at the N-terminus. AtRidA-S68 crystal structure is disordered in the first 12 residues, which accounts for the missing strand. We also prepared a construct that starts with V57, but this construct did not behave well and no crystals could be obtained. Structural analyses indicate that this strand interferes with the packing pattern and thus the crystallizability of the protein (data not shown).

### Trimer formation

**Figure 2:**
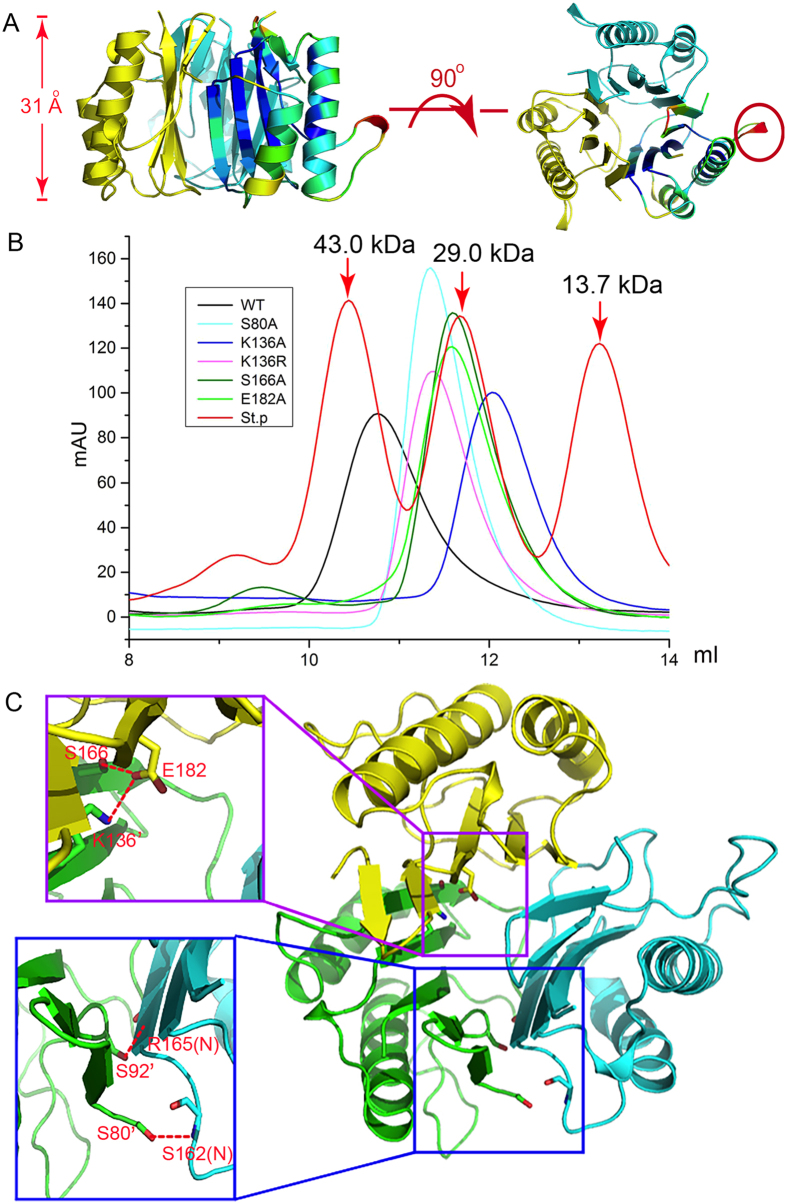
The overall structure the RidA trimer. (**A**) Two orthogonal views of the trimer. One of the monomers is colored according to the regional B-factors of the protein. The fragment that has high B-factors is indicated by the circle. (**B**) The oligomeric states of the WT RidA and mutants as assessed by size-exclusion chromatography. 0.5 mg of RidA WT and mutants were loaded onto a Superdex-75 size-exclusion column separately. Protein standards were used as a size reference and their respective sizes are indicated by the numbers on the top of each peak (red trace, St. p). (**C**) The interaction network that maintains the trimer assembly from top view. The insets show close-up views of the interface and primes denote residues from the neighboring subunits. The possible hydrogen bonds and salt bridges are represented by the red dashed lines (cutoff distances are set to 3.3 Å).

The space group of apo-AtRidA is H3 and through symmetry operation, a homotrimer can be generated. There is a central cavity in the barrel-like trimeric structure, whose height is ~31 Å (Fig. 2A). The inner side of the barrel is formed by the β-sheet from each monomer, and the bottom is sealed by the Q169-K178 loop. The α-helices pack on the outside of the barrel, while the β-strands are approximately parallel to the 3-fold crystallographic axis. The buried surface between the neighboring monomers is 1306.2 Å^2^. A small fragment P99-T101 is at the distal end of the trimer structure, and has higher B-factors than other regions (Fig. 2A).

RidA exists as a homotrimer not only in crystals, but also in solution. The protein shows a peak at 10.7 ml on a Superdex-75 size-exclusion column and corresponds to a molecular weight of ~40 kDa, roughly the size of a trimer (Fig. 2B). From sequence alignment and structure superposition, we discovered that two invariant residues, K136 and E182, are critical for the interfacial interactions by forming a monodendate salt bridge (Fig. 2C). Additionally, E182 also makes a hydrogen bond with the highly conserved residue S166, further reinforcing the interactions between the neighboring monomers. Furthermore, two serine residues, S80 and S92 are involved in hydrogen-bonding interactions with the backbone nitrogens of S162 and R165 respectively. To test their contribution to the trimer assembly, we mutated these residues to Ala or Arg (S80A, S92A, K136A, K136R, S166A and E182A) by site-directed mutagenesis. All these mutations did not significantly reduce the stability of AtRidA except for the S92A mutant, which is prone to precipitation. Size-exclusion chromatography analyses showed that all these mutants have slower migration rates than that of WT, suggesting partial disruption of the trimer. K136R and S80A affect the interface the least, while K136A severely impacts the assembly, which underscores the indispensable role of the K136-E182 salt bridge as well as the inter-subunit hydrogen bonds in maintaining the oligomeric state (Fig. 2B).

### Complex structure with pyruvate

In addition to the apoprotein structure, we also tried to obtain the complex structures of AtRidA as well, in order to further explore its ligand recognition mechanism. Threonine dehydrogenase is able to remove a water molecule from both serine and threonine to generate the imine intermediates, which are in turn converted to 2-oxoacids (2-oxobutanoate or pyruvate) by the deamination activities of RidA. The intermediates are highly unstable and have short lifetimes, which are unsuitable for crystallization. Therefore we tried both 2-oxoacid products for cocrystallization studies, and we obtained the complex structure of AtRidA bound by pyruvate.

**Figure 3:**
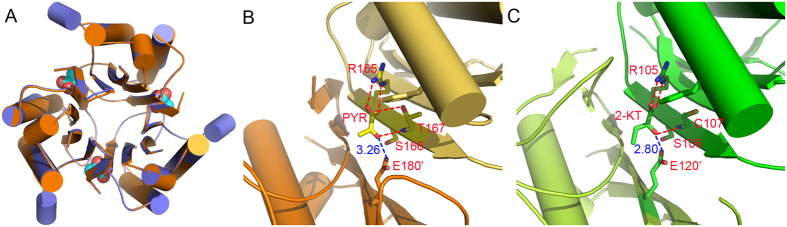
The ligand recognition pattern by RidA. (**A**) The superposition of the trimer AtRidA structure with (orange) and without (violet) pyruvate. The pyruvate ligands are shown as the spheres. (**B**) The specific recognition of pyruvate by AtRidA; (**C**) and 2-oxobutanoate by Tdcf (Hydrogen bond cutoff distances are set to 3.3 Å). The blue lines show the distances between the conserved glutamate and the ligand carbonyl groups.

The structure was obtained by soaking the crystals of the apoprotein with 20 mM pyruvate for 1 hour, and the resulting crystals diffracted to 2.3 Å. The crystals did not appear to have decayed, but the crystal space group became *P*1. After structure determination, we found that there were three monomers present in the asymmetric unit. In each monomer of the final model, residues S80-L187 are resolved without internal disorder. The three monomers within the asymmetric unit form the trimeric barrel, which is almost identical to that formed by the apoprotein, with an RMSD of only 0.27 Å (Fig. 3A). Pyruvate binds to the interfacial cleft formed by two subunits, but makes specific hydrogen bonds with only one subunit. The carboxylate group of pyruvate forms the salt bridge with the NH2 and NE atoms of the guanidino group of conserved R165, while accepting another from the OG atom of T167. Additionally, O3 of the carbonyl group receives another hydrogen bond from the backbone of T167 (Fig. 3B). Aside from these polar contacts, the ligand is located at the active site formed by F147, L95 and P174. When superposing with the structure of TdcF complexed with 2-oxobutanoate, we discovered that the binding modes of these 2-oxoacids are conserved. The only difference is that in the latter, the interaction with the threonine is lost, due to the substitution of T167 with a cysteine in TdcF (Fig. 3C). We also tried 2-oxobutanoate as a ligand for crystallization, but the four datasets we collected invariantly showed incomplete electron density of 2-oxobutanoate, regardless of how we obtained the crystals of the complex. A closely related compound 3-oxobutanoate is known to undergo decarboxylation to generate acetone on heating under acidic conditions, but the quick breakdown of 2-oxobutanoate is unlikely. We think that a lack of specific contacts on the C3 and C4 atoms results in a relatively flexible tail of the ligand, and leads to partial electron density.

Although we could not obtain the structure of AtRidA in complex with the enamine/imine substrates, we can still derive useful information from the structure of the AtRidA-pyruvate complex. The only difference between the enamine/imine substrates and the 2-oxoacids is the substitution of the imine at position C2 by a carbonyl group (Fig. 1A). Therefore, the same interaction between the T167 backbone and the 2-carbonyl group can be preserved during the substrate recognition process by the enzyme, due to the same sp^2^ hybridization of C2. Additionally, the terminal carboxylate of E180 from the neighboring subunit is only 3.26 Å from the 2-carbonyl group, and hence it is very likely to play a role in the recognition of the intermediates.

### Enzyme Assays

**Figure 4:**
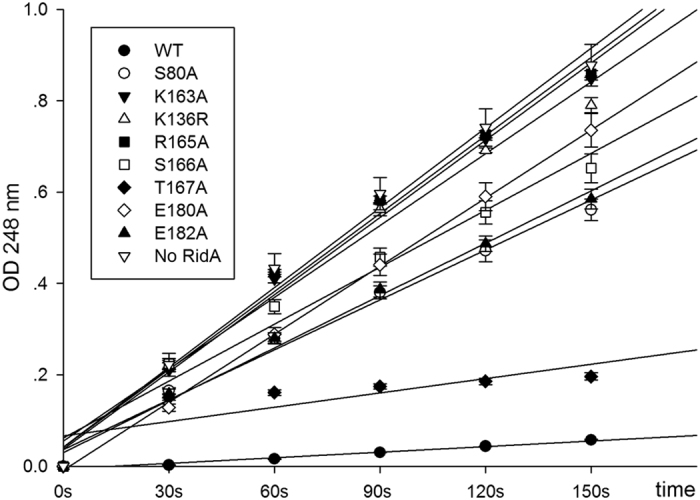
The enzymatic activity assays of WT RidA and mutants. The rate of semicarbazone (Sc) formation was measured by absorbance at 248 nm, and the assay mixtures contained 10 μM RidA WT and mutants. Semicarbazone formation is slowest in the presence of WT RidA because the enzyme removes most of the reactive imine before it has a chance to react with semicarbazide. Greater rates of semicarbazone formation are noted in the presence of the mutants because the mutant enzymes are unable to hydrolyze the imine as effectively as the WT enzyme.

To assess the importance of individual residues to enzyme activities, we measured the activities of RidA and mutant proteins by monitoring the UV-absorbance of semicarbazones, which are the reaction products of semicarbazide with the imines^24^. The deamination activity of RidA converts the imine intermediates to 2-oxoacids, which can be further reduced by lactate dehydrogenase in the presence of NADH. The reactions are started by threonine in the absence of RidA, at which time the synthesis of semicarbazones proceeds at the maximal rate (Fig. 4). The mutants we tested not only include the residues that are directly involved in substrate/product binding, but also the residues responsible for the trimer formation.

The addition of 10 μM RidA WT reduced semicarbazone formation over a time course of 150 s, where the initial velocity of the enzyme remains linear. By contrast, the mutations reduced the deamination rates by various degrees. R165A, K136A/R are among the mutations that caused the most reduction in enzymatic activities, with the R165A mutant only retaining 2.2% activity (Fig. 4). The greatest loss of enzyme activity by the R165A mutation was consistent with the previous report that a RidA-deficient (ΔridA) *Salmonella enterica* strain transformed with the R165A mutant from *Arabidopsis thaliana* completely failed to complement the activity^16^. On the other hand, E180A, E182A, S80A and S166A only caused moderate decreases, with at least one-quarter of activity remaining. T167 is a non-conserved residue, and the T167A mutant still retains ~80% activity. These results suggested that the trimer assembly also plays a role in the deamination activities of RidA. In addition, the moderate reduction of the E180A mutant suggests possible interactions between E180 and the nitrogen atom of the imine substrates.

RidA is an important enzyme despite its small size. *In-vitro* activity assays indicate that it is a cofactor-independent deaminase. Well-characterized deaminases such as adenosine deaminases have been found to play an important role in RNA editing, and utilize RNAs as substrates. These enzymes are usually zinc-dependent metalloenzymes, in which the metal ion coordinates and activates a water molecule for catalysis. By contrast, RidA appears to employ a distinct catalytic mechanism and none of the biochemical experiments or crystal structures to date indicated that RidA relies on metal ions or cofactors for catalysis. Based on modeling studies, Lambrecht *et al.* proposed a mechanism for RidA, in which the water molecule coordinated by the enzyme near the enamine/imine acts as a nucleophile^8^, but more supporting evidence is certainly needed to investigate the mechanism of this interesting enzyme.

Taken together, we present the first crystal structure of the RidA protein from plants. The RidA domain retains the basic overall fold, and also preserves the homotrimeric structure with the characteristic interfacial clefts. In addition, a complex with pyruvate suggests that R165 and T167 play key roles in the recognition of the carboxylate group, and may also be responsible for the recognition of the imine substrate using the same type of interactions. Lastly, through activity assays, we discovered that the trimeric state is equally important to enzymatic activities, to which K136 and R165 contribute the most. This study provides structural insight into RidA functions in plants.

## Experimental Procedures

### Cloning, expression and purification of RidA

The full-length *Arabidopsis thaliana* RidA gene (GenBank accession No. AY060547.1) was amplified from *Arabidopsis* cDNA. After double digestion by the *Nde*I and *Xho*I restriction enzymes, the PCR product was ligated into a modified pET-28a (+) vector (Novagen) vector with an engineered PreScission protease cleavage site (recognition peptide LEVLFQGP) preceding the His6-tag. For crystallization purposes, N-terminal truncated fragments with various lengths were cloned into pET-28a (+) using the same sites. The primers used in this study were shown in Table 1. These plasmids were transformed into the *Escherichia coli* strain BL21 (DE3) cells. To overexpress the protein, 10 ml overnight culture was used to inoculate 1 L fresh culture medium containing 30 μg/ml kanamycin and grown at 37 °C until the log phase was reached (OD_600_ = 0.8). The expression of RidA-S68 was induced by isopropyl β-D-1-thiogalactopyranoside (IPTG) at a final concentration of 0.2 mM, at 25 °C for 18 hours.

Cells were harvested by centrifugation at 3,030 g for 20 min at 4 °C, and resuspended in pre-chilled nickel-nitrilotriacetic acid (Ni–NTA) buffer A (250 mM NaCl, 10 mM imidazole, 40 mM Tris-HCl, pH 8.0, 1 mM phenylmethylsulfonyl fluoride (PMSF) and 1 mM β-mercaptoethanol) before lysis. The cells were disrupted by sonication, and the supernatant was collected by centrifugation at 23,500 g for 60 min at 4 °C. The supernatant was mixed with the pre-equilibrated Ni–NTA affinity resin (Qiagen) at 4 °C for 1 h. The bound protein was washed with 10 column volumes of buffer A, and the target protein was then eluted with Ni-NTA buffer B, which contains the same components of buffer A plus 250 mM imidazole. The his6-tag at the N-terminus was cleaved off by treating with the PreScission protease overnight in the presence of 5 mM β-mercaptoethanol, and the reaction mix was subsequently applied onto a Histrap column (GE Healthcare) to remove uncut protein. The unbound portion was pooled, dialyzed to a buffer containing 20 mM Tris-HCl (pH 8.0), 150 mM NaCl and 1 mM DTT for two hours. The protein was further concentrated to 10 mg/ml, then flash frozen in liquid nitrogen and stored at −80 °C.

All point mutations for RidA activity assays were created by QuikChange. The RidA-S68/pET28a (+) plasmid (the fragment represents residues S68–L187) was used as the template. All mutations were confirmed by DNA sequencing and the integrity of the expressed proteins was assayed by a Superdex 75 size-exclusion column. 10% (v/v) glycerol was added to the purified mutants before they were flash frozen. The cloning, expression and purification of *Arabidopsis thaliana* threonine dehydratase was described in a previous reference^16^.

### Crystallization and data collection

Among the several constructs we tested, the RidA-S68 construct allowed us to produce diffracting crystals. Crystals were obtained using the sitting drop vapor diffusion method in a 96-well plate after ten days at 25 °C. The reservoir solution contains 18% PEG 3350, 0.1 M MES, pH 6.0. The crystals of RidA with ligand pyruvate were obtained by soaking the crystals of the apoprotein with 20 mM pyruvate for 1 hour. The best crystals were transferred to a cryo-protectant solution (20% (v/v) glycerol plus the reservoir solution) and were flash frozen in liquid nitrogen. Native data for AtRidA in the apo form or in complex with pyruvate were collected from frozen crystals at −173 °C using an in-house Oxford Diffraction Xcalibur Nova diffractometer. The data were processed and scaled using CrysAlisPro (v.1.171.33.49; Oxford Diffraction) and SCALA from the CCP4 suite^25^. The space group of the crystals for apoprotein was *H*3 and the crystal diffracted to 2.0 Å resolution with a completeness of 99.87%. Cell content analysis suggested that each asymmetric unit contains one monomer. To solve the structure, coordinates of a putative endoribonuclease (TM0215) structure (PDB entry 2B33) were used as the search model for molecular replacement. The initial model was extended by PHENIX using the autobuild option^26^, and the resulting model was further built manually according to the electron-density map with Coot^27^. Multiple cycles of refinement alternating with model rebuilding were carried out by REFMAC5^28^. The R factor was 15.3% (R_free_ = 21.2%) for the final model, which was validated by SFCHECK and Molprobity^29^,^30^. The Ramachandran plots of the final models have 98.1%, 1.9%, 0% residues in the most favorable, generously allowed and disallowed region for the apoenzyme. The structure of the complex with pyruvate was solved using the apo protein structure and the space group was *P*1. The crystal diffracted to 2.3 Å resolution and each asymmetric unit contains three monomers. The R factor was 20.4% (R_free_ = 23.8%) for the final model. The Ramachandran plots of the final models have 96.2%, 3.8%, 0% residues in the most favorable, generously allowed and disallowed region for the apoenzyme. The data collection and refinement statistics are listed in Table 2. All figures were produced with PyMOL (http://www.pymol.org). The secondary structure of RidA-S68 was prepared by ESPript^31^.

### Enzyme activity assays

The activity assays followed a previous protocol described by Niehaus *et al.*^16^. Briefly, a 500-μL assay mixture contains 50 mM potassium pyrophosphate, pH 8.7, 10 mM semicarbazide-HCl (neutralized), 0.5 μM threonine dehydratase, and 10 μM of RidA. 2 mM L-Thr (final concentration) is added to the assay to start the reactions, and the absorbance values at the time point 90 s are used to calculate the relative activity with the formula: the relative activity = (A_no RidA_ − A_mut_)**/**(A_no RidA_ − A_RidA WT_) * 100%, where A_no RidA_, A_RidA_ and A_RidA WT_ are the absorbance values at 248 nm in the absence of the enzyme, in the presence of the RidA mutants and the RidA WT at the time point 90 s, respectively.

## Additional Information

**Accession codes:** The atomic coordinates and structure factors have been deposited in the Protein Data Bank with accession numbers 5HP7 and 5HP8 for the apo and complex structure of AtRidA respectively.

**How to cite this article**: Liu, X. *et al.* Crystal structures of RidA, an important enzyme for the prevention of toxic side products. *Sci. Rep.* **6**, 30494; doi: 10.1038/srep30494 (2016).

## Figures and Tables

**Table 1:** The primers used in this study.

Name	Sequence
F-RidA -FL-NdeI	AATAGATTCATATGACTTGGTCGGTTTTCAGA
R-RidA-FL-XhoI	TTGCACTTCTCGAGCTAGAGTGTTGCAATACATTCAAT
F-RidA-S68-NdeI	AATAGATTCATATGTCTACTGAGAAAGCACCAGC
F-RidA-S80A	GCTTTGGGACCTTA*CGC*TCAGGCCATTAAAG
R-RidA-S80A	CTTTAATGGCCTGA*GCG*TAAGGTCCCAAAGC
F-RidA-S92A	TCTGGTTTTTCTT*GCA*GGTGTTCTTGG
R-RidA-S92A	CCAAGAACACC*TGC*AAGAAAAACCAGA
F-RidA-K136R	TCCTCGGTGGTG*AGG*ACAACAATCATG
R-RidA-K136R	CATGATTGTTGT*CCT*CACCACCGAGGA
F-RidA-K136A	ATTCCTCGGTGGTG*GCG*ACAACAATCATGT
R-RidA-K136A	ACATGATTGTTGT*CGC*CACCACCGAGGAAT
F-RidA-R165 A	CTCCTTCTCCAGCA*GCA*TCGACGTATCAAG
R-RidA-R165 A	CTTGATACGTCGA*TGC*TGCTGGAGAAGGAG
F-RidA-S166A	CCTTCTCCAGCACGA*GCG*ACGTATCAAGTTG
R-RidA-S166A	CAACTTGATACGT*CGC*TCGTGCTGGAGAAGG
F-RidA-T167A	CCAGCACGATCG*GCG*TATCAAGTTG
R-RidA-T167A	CAACTTGATA*CGC*CGATCGTGCTGG
F-RidA-E180A	AAACGCCAAGATC*GCG*ATTGAATGTATTG
R-RidA-E180A	CAATACATTCAAT*CGC*GATCTTGGCGTTT
F-RidA-E182A	CAAGATCGAGATT*GCA*TGTATTGCAACAC
R-RidA-E182A	GTGTTGCAATACA*TGC*AATCTCGATCTTG

The restriction sites were underlined in the amplifying primers while the mutated bases were italized in the *Quikchange* primers.

**Table 2:** Data collection and refinement statistics.

Crystals	Apo-RidA (5HP7)	RidA-pyruvate complex (5HP8)
Data collection		
Wavelength (Å)	1.54 Å	1.54 Å
Space group	*H*3	*P*1
Cell dimensions (Å)		
a, b, c (Å)	76.9, 76.9, 40.3	40.3, 46.3, 46.4
α, β, γ (°)	90.0, 90.0, 120.0	111.4, 107.7, 106.3
Resolution (Å)	25.67–2.00 (2.11–2.00)^a^	22.30–2.30 (2.42–2.30)
R_merge_^b^	0.045 (0.183)	0.065 (0.276)
R_*meas*_	0.055 (0.227)	0.086 (0.374)
CC_1/2_	0.998 (0.949)	0.997 (0.912)
Wilson B	19.3	34.8
I/σ_(I)_	16.2 (5.5)	8.2 (2.5)
Completeness (%)	99.9 (99.3)	99.30 (98.4)
Redundancy	3.0 (2.9)	1.9 (1.9)
Refinement		
Resolution (Å)	25.67–2.00 (2.05–2.00)	22.30–2.30 (2.53–2.30)
No. reflections	5725	11771
R_work_^c^/R_free_^d^	0.153/0.212	0.204/0.238
No. atoms		
Protein	808	2424
Ligand	—	18
Water molecules	85	36
B-factors (Å ^2^)		
protein	19.4	31.0
Ligand	—	33.3
Water	30.7	31.3
R.m.s deviations		
Bond lengths (Å)	0.016	0.003
Bond angles (°)	1.46	0.82

^a^Values in parentheses are for the highest-resolution shell.

^b^R_merge_ = Σ |(I − < I > )|/σ(I), where I is the observed intensity.

^c^R_work_ = Σ_hkl_ ||Fo| − |Fc||/ Σ_hkl_ |Fo|, calculated from working data set.

^d^R_free_ is calculated from 5.0% of data randomly chosen and not included in refinement.
